# Agent-oriented Decision Support System for Business Processes Management with Genetic Algorithm Optimization: an Application in Healthcare

**DOI:** 10.1007/s10916-020-01608-4

**Published:** 2020-08-02

**Authors:** Emilio Sulis, Pietro Terna, Antonio Di Leva, Guido Boella, Adriana Boccuzzi

**Affiliations:** 1 Computer Science Department, University of Torino, Torino, Italy; 2 University of Torino, Torino, Italy; 3 Azienda Ospedaliero Universitaria San Luigi Gonzaga, Orbassano, Italy

**Keywords:** Agent-based decision support system, Emergency department planning, Genetic algorithm, Business process management

## Abstract

Agent-based approaches have been known to be appropriate as systems and methods in medical administration in recent years. The increased attention to processes led to the recent growth of Business Process Management discipline, which quite exclusively adopt discrete-event modeling and simulation. This paper proposes a medical agent-oriented decision support system to integrate the achievements from management science, agent-based modeling, and artificial intelligence. In particular, we performed a practical application concerning a hospital emergency department medical system. We adopt the widely used multi-agent programmable modeling environment NetLogo. First, we demonstrated the ability to perform a clear representation of healthcare processes where agents (i.e., patients and hospital staff) operate in a 3D environment. This model allows performing a traditional *what-if* scenario analysis. Second, we explore how performing intelligent management of patients by applying genetic algorithms to find the criteria for the selection process of the subjects in the admission procedure. The results are encouraging towards a more extensive application of agent-oriented methodologies in healthcare management.

## Introduction

This paper proposes the adoption of an agent-oriented approach to investigate the organizational level in the healthcare business process. Agent-based methodologies already demonstrated their validity in healthcare practical applications [14]. In recent years, the growing interest in business process analysis in management science [6] mostly resulted in researches quite exclusively focused on a discrete event modeling perspective [23], where events occur in a time-stepped simulation. Therefore, modeling and simulation mostly refer to the investigation of scenario and *what-if* analysis, prediction of the immediate or short-term next behavior of the system [1], playing a key role in addressing management [4, 7]. Nevertheless, agent-oriented computational models demonstrated their ability to simulate actions and interactions of autonomous agents, with the primary goal of assessing their effects on the system as a whole. In Agent-Based Modeling (ABM) the focus is on emergent phenomena [5] in complex adaptive systems [11]. Some efforts focused on the interaction between individual behavior and the environment [17], initially exploring the topic of business processes [16]. In contrast, ABMs can “provide a more fine-grained model of the process, with many parameters that can impact the dynamics. We call such models, which explicitly model the individual agents, agent-based simulation models” [20].

This work is an attempt to reduce the gap between between the discipline of Business Process Management (BPM) and agent-based methodologies by proposing a real application in the healthcare domain. In particular, we apply an agent-oriented framework to investigate the organization of an Emergency Department (ED), one specific type of healthcare business process. We adopted the open-source multi-agent programmable modeling environment NetLogo, a sort of benchmark tool-kit largely adopted in several disciplines [25]. Business process analysis traditionally considers specific formalisms, e.g., Directly-Follows graphs, Petri Nets, Business Process Modeling Notation (BPMN) [3]. Healthcare stakeholders include doctors and nurses who miss the training to understand such modeling formalism. So, we paid attention to adopt a clear visualization of agents acting in the environment, by considering how agent-oriented approaches perfectly fit this need, as the concept of the agent is understandable also for not expert specialists of AI domain [19]. In a first part of our work, we propose an healthcare agent-based model that can be used to explore scenario analysis in the framework of BPM. Second, we explore an agent-oriented decision support system by considering the variation of parameters (parameter sweeping), towards their optimization with genetic algorithms (GA) [18]. To both minimize the length of stay of patients and maximize the throughput, paying attention to standard quality in the framework of ED regulations, we explore a five-dimensional space. By applying stochastic optimization technique to agents interactions, we demonstrate the feasibility of this kind of agent-oriented medical decision support system. The paper is structured as follows: “Background” describes related work and case study, while we discuss the methodological framework with the output of the modeling phase in “Modeling and simulating the Emergency Department”. We adopted the typical Overview Design Detail protocol (ODD) [10], largely used to introduce agent-based models presenting the model results. Section “Admission process and genetic algorithm” describes parameter sweeping and GA results, while “Conclusions” concludes the paper.

## Background

In the context of agent-based applications to healthcare [2], recent efforts focused on the role of autonomous agents and multi-agent systems in healthcare [15]. As for agent-based simulations, most covered topics were logistic and marketing [9]. Moreover, agent-based modeling has been applied in healthcare domain [8], mostly in “agent-based care platforms and simulation” (21.8 %). Instead, the topics covered in our work are less frequent, i.e., “decision support systems” is about 11.3 %, and planning 8.3 % [12]. Recent works focused on Emergency Department simulation [13] also for operational management [21, 22]. An interesting perspective is the adoption of Artificial Intelligence (AI) techniques to ABM in BPM.

### Case study

Our work refers to a hospital ED in a densely populated area in Northern Italy.^1^ The department includes a staff of seven ED nurses as well as four doctors, two triage nurses, and two social workers. The services (or exams) provided by the department are blood analysis, radiology, and imaging tests. The total number of patients is around 47,000 in one year, for a daily average of about 125 cases. The distribution of patients arrivals varies according to the day of the week: Monday and Friday are the peak days (16.9% and 15.3% in 2019), while lowest frequencies occur on Saturday and Sunday (12.4% and 11.4%). The arrival rate on Tuesday, Wednesday, and Thursday is 15%, 14.4%, and 14.6%, respectively. The Emergency Severity Index (ESI) adopted in Italy since 2019 was a four-level scale of urgency ranging from 1 (very high) to 4 (very low). From 2020 Italian regulations introduced the international five-level scale. The sequence of activities in ED is quite standard. While urgent cases are taken directly into Shock-Room, other patients follow the Registration-Triage-Visit path, possibly including the need for exams or medical consultancies. Managers of the hospital department are interested in better understand the ED model to perform some changes in the organization (working hours of the operators, sequence of activities, services scheduling).

## Modeling and simulating the Emergency Department

### Overview

**Fig. 1:**
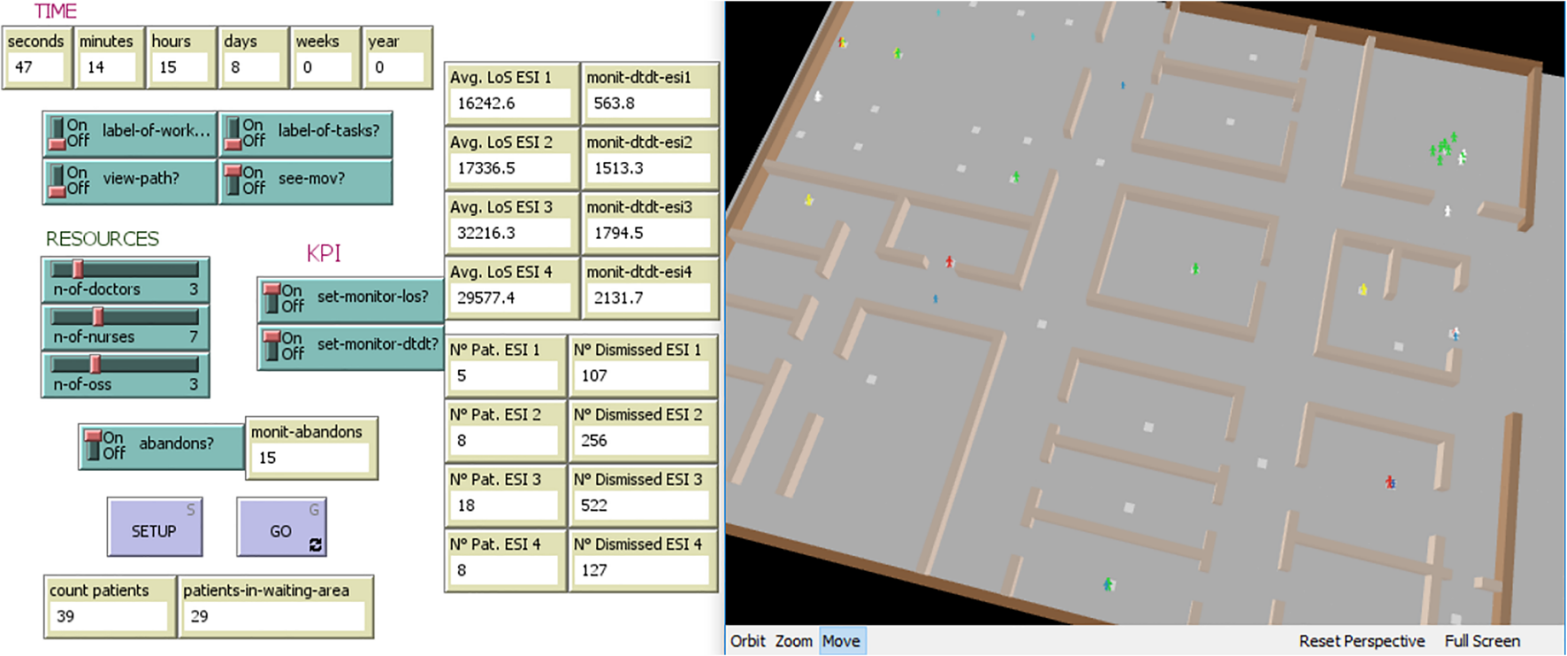
Interface with buttons, monitors and the output area in a 3D version of the Emergency Department to better appreciate operators and patients movements

Our interest here is in exploring agent-based modeling of a business process. Agents act in a 3D environment based on the department map to implement flows between different activities (Fig. 1). Agents follow rather simple behavioral rules, according to their state: at first, they look for the next patient, then move to the activity, waiting to start, working on the task, and finally looking for the next patient. Activities are objects which include variables of interest (e.g., number and type of workers, average and standard deviation of duration). Agents interact with other agents and activities/environment. We define the paths through a node graph (each node is an activity). The weight of the arc is the average duration of the walk between the two nodes. This agent-oriented simulation includes characteristics on the workers, such as their skills/speed of execution (e.g., distinguishing between experts or beginners, with a different degree of work ability).

At the beginning of the simulation, each agent moves according to their state. Once an activity is free, workers select the patient with the highest priority (defined by criteria considering both their urgency level and waiting time). They start working on the task for the time specified by the *Duration* agents’ variable. At the end of the time slot, patients update their *next-task* variable with the name of the next activity and move to the waiting area until the next task becomes available.

### Design

The model proposes to shape the general functioning of the ED emerging from the simple behavioral rules of agents. Free operators select the next patient on (i) the priority of urgency and (ii) the time already spent in the ED. As some patients may decide to abandon the department before being seen by a doctor, we consider several variables of the agent, including their urgency level (very low or low), the number of other agents currently in the waiting area, the time spent from the arrival in the ED. Stochasticity is relevant to represent the arrival of patients each day. We set a random number of patients each day to obtain different values, but always according to real distribution. Similarly we modeled the total number of arrivals for each day (e.g., Monday is different from Sunday).

### Details

The initial state of the model corresponds to the empty department at midnight on Monday. To correctly manage business process indicators, we consider a warm-up period of one day. We import the path in the ED from an external file of the network in graphml format. Each node includes the following information: name of the activity, number of operators needed, type of operator(s) required, duration of the work for each operator.

### Performance Process Indicators

To compare simulation results and to test their validity, in the literature [24] exist two leading indicators. They are the Length of Stay (LoS), which is the average time spent by each patient from the arrival to the discharge, and the Door-to-Doctor-Time (DTDT), i.e., the time between admission and the first visit of a doctor.

### Results

We compute the average value and the standard deviation of our two leading performance indicators (see Table 1) by the model, ten times for a period of four weeks. The average DTDT depends on the patient’s urgency level, from 11 to 13 minutes, with small variability. Similar works obtained a range of “13 minutes to 30 minutes with mean and median times of 24.28 and 20 minutes” [13]. The indicator concerning patient throughput time (LoS) is about 3.5 hours (219.1 minutes) for most critics patients and up to more than 7 hours (433.6 minutes) for less critics ones. ED managers consider these values generally quite realistic (at most slightly underestimated), as well as in line with values in the literature [24].

**Table 1:** Simulation output of two leading performance indicators (Door-to-Doctor-Time, DTDT and Length-of-Stay, LOS) in four weeks, patients by ESI (times in minutes)

Performance Indicator		ESI1	ESI2	ESI3	ESI4
DTDT	Avg	11.2	16.9	21.1	22.7
	St.Dev.	0.8	0.1	0.6	1.3
LOS	Avg	219.1	258.2	430.3	433.6
	St.Dev.	19.1	11.8	6.8	23.2

#### Resource utilization

In addition to performance metrics, a particular interest refers to the utilization of resources. In our model, we compute two indicators concerning the working time of doctors and nurses. The rate of minutes worked by doctors directly with patients over the four weeks of the simulation is about 83.5%, while the corresponding percentage for nurses is 46.2%. The validity is confirmed by hospital staff, as nurses have several tasks not strictly related to patients (e.g., manage drugs, prepare tools, talk to relatives etc.).

## Admission process and genetic algorithm

### Patient registration process

**Fig. 2:**
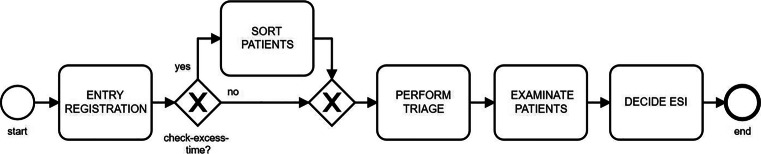
Triage and registration process in BPMN

In addition to the general functioning of the process, a relevant problem for management concerns patient scheduling. We explored the adoption of GA technique with the tasks in Fig. 2. The two main selection criteria of the next patient are the urgency level and the waiting-time. If we give the priority always to urgency, the not urgent cases could wait an excessive time; on the contrary, giving priority exclusively on arrival order has the risk of not treating severe cases in time. We propose a formula to evaluate the solutions, paying attention to the fact that more urgent cases (ESI 1) must be immediately take in charge. Nevertheless, less urgent cases can be procrastinated but not over a certain threshold. The *check-excess-time* gateway allows to sort patients in queue taking into account these thresholds. Accordingly to our domain experts, we compute a Quality Score (QS) as the square of both the number of urgent cases and less critical patients served under a certain threshold. Thus, we can evaluate model results.

### Parameter sweeping

**Fig. 3:**
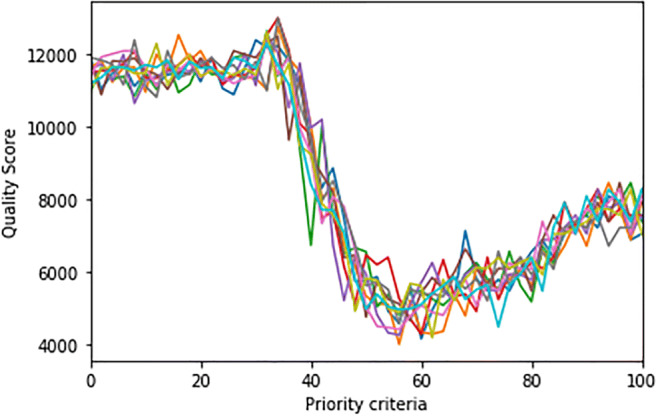
Parameter sweeping results of ten runs by varying “priority-criteria” to minimize QS

To investigate parameter sweeping, we used a software tool integrated into NetLogo (BehaviorSpace).^2^ First, we find the value of a parameter minimizing the QS value, just considering the selection criteria by varying the probability to select the next patient by urgency or by waiting-time. We model this decision made by free workers when they have to choose the patient as a probability expressed in percentage, called “priority-criteria.” Our model executed systematically varying the settings of interest and recording the results of each run. The exploration of the model’s “space” of possible behaviors determines which combinations of settings cause the responses of interest. We noticed how the critical “priority-criteria” parameter produces a U-shaped curve (Fig. 3), suggesting that the value of the parameter minimizing QS is between 55 and 60.

### Genetic algorithm results

In a second step, we consider a more sophisticated solution. To test the construction of the GA we face first of a well-defined problem, already having the brute force solution. We adopted an external tool linked to NetLogo.^3^ In our first experiment, we set a population size of 50, a crossover rate of 0.7, a mutation rate of 0.3. The goal is to minimize QS only by varying the criteria in a range of 5 between 0 to 100. The QS value obtained with GA is 60 with a fitness value of 4,373, allowing us to assess the goodness of our model being very similar to the value obtained by parameter sweeping.

Once validated the model, a more interesting research topic relates finding the maximum waiting parameters (measured in seconds) for each kind of urgency (from ESI 2 to ESI 5, as urgent cases ESI 1 are immediately treated), besides the “priority-criteria” selection parameter, in a range of 5 between 0 to 100. The four ESI thresholds vary in the following ranges: threshold-ESI2 [900-3,600], threshold-ESI3 [1,800-5,400], threshold-ESI4 [3,600-9,600], threshold-ESI5 [7,200-14,400].

**Fig. 4:**
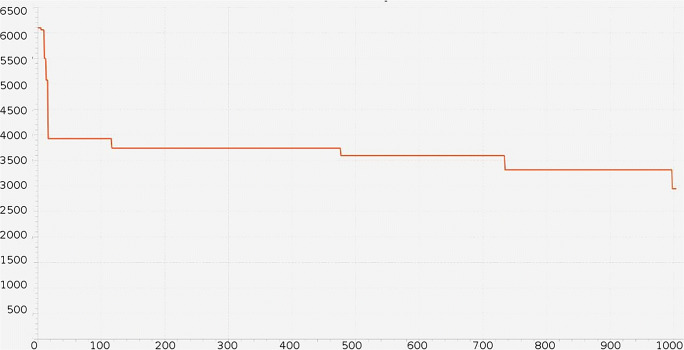
GA fitness Quality Score reaches a minimum value of 2,941, greatly improving the value obtained with the brute-force approach

Hence, the space to investigate becomes five-dimensional. GAs always look for the minimum of QS, with an initial population size of 100, a crossover rate of 0.7, a mutation rate of 0.3. Finally, GA results are really of interest with a fitness value of 2,941 (See Fig. 4), obtained with the following five values that give that minimum: priority-criteria: 55; threshold-ESI2: 1,380; threshold-ESI3: 2,280; threshold-ESI4: 9,060; threshold-ESI5: 7,200.

## Conclusions

This work discussed the adoption of an agent-based modeling approach to a healthcare process to address hospital management. The issues solved are typical of business process analysis by considering a set of performance indicators and scenario analysis. To investigate patient scheduling as an optimization problem searching the parameter-space, we applied the AI technique of GA. In our work, GA evolving the parameters determining the behavior of a whole system, which we represent via an ABM. In the proposed approach, once the model has been validated, we initially compared parameter sweeping and GA. As a brute-force approach finds the same solution of GA with a single parameter, this confirms of the correct setting of the GA. Then we performed an exploration of a five dimensional space problem to find the thresholds (i.e., the maximum waiting parameters set by type of urgency) that can be suggested for medical reasons to select the next patient in the admission process. GA results suggest parameter values to decision-making in order to improve the quality of the process, accordingly to criteria defined by medical management.

## Footnotes

1 Cfr. San Luigi Hospital, Orbassano, Torino, Italy http://www.sanluigi.piemonte.it/info/index.shtml

2 Cfr. https://ccl.northwestern.edu/netlogo/docs/behaviorspace.html

3 Cfr. BehaviourSearch http://www.behaviorsearch.org/
